# Divergence in Population Coding for Space between Dorsal and Ventral CA1

**DOI:** 10.1523/ENEURO.0211-21.2021

**Published:** 2021-09-07

**Authors:** Udaysankar Chockanathan, Krishnan Padmanabhan

**Affiliations:** 1 Neuroscience Graduate Program, University of Rochester School of Medicine and Dentistry, Rochester, NY 14623; 2 Medical Scientist Training Program, University of Rochester School of Medicine and Dentistry, Rochester, NY 14623; 3 Ernest J. Del Monte Institute for Neuroscience, University of Rochester School of Medicine and Dentistry, Rochester, NY 14623; 4 Department of Neuroscience, University of Rochester School of Medicine and Dentistry, Rochester, NY 14623; 5 Center for Visual Science, University of Rochester School of Medicine and Dentistry, Rochester, NY 14623

**Keywords:** correlations, dCA1, hippocampus, maximum entropy models, neural coding, vCA1

## Abstract

Molecular, anatomic, and behavioral studies show that the hippocampus is structurally and functionally heterogeneous, with dorsal hippocampus implicated in mnemonic processes and spatial navigation and ventral hippocampus involved in affective processes. By performing electrophysiological recordings of large neuronal populations in dorsal and ventral CA1 in head-fixed mice navigating a virtual environment, we found that this diversity resulted in different strategies for population coding of space. Populations of neurons in dorsal CA1 showed more complex patterns of activity, which resulted in a higher dimensionality of neural representations that translated to more information being encoded, as compared ensembles in vCA1. Furthermore, a pairwise maximum entropy model was better at predicting the structure of these global patterns of activity in ventral CA1 as compared with dorsal CA1. Taken together, the different coding strategies we uncovered likely emerge from anatomic and physiological differences along the longitudinal axis of hippocampus and that may, in turn, underpin the divergent ethological roles of dorsal and ventral CA1.

## Visual Abstract

**Figure:**
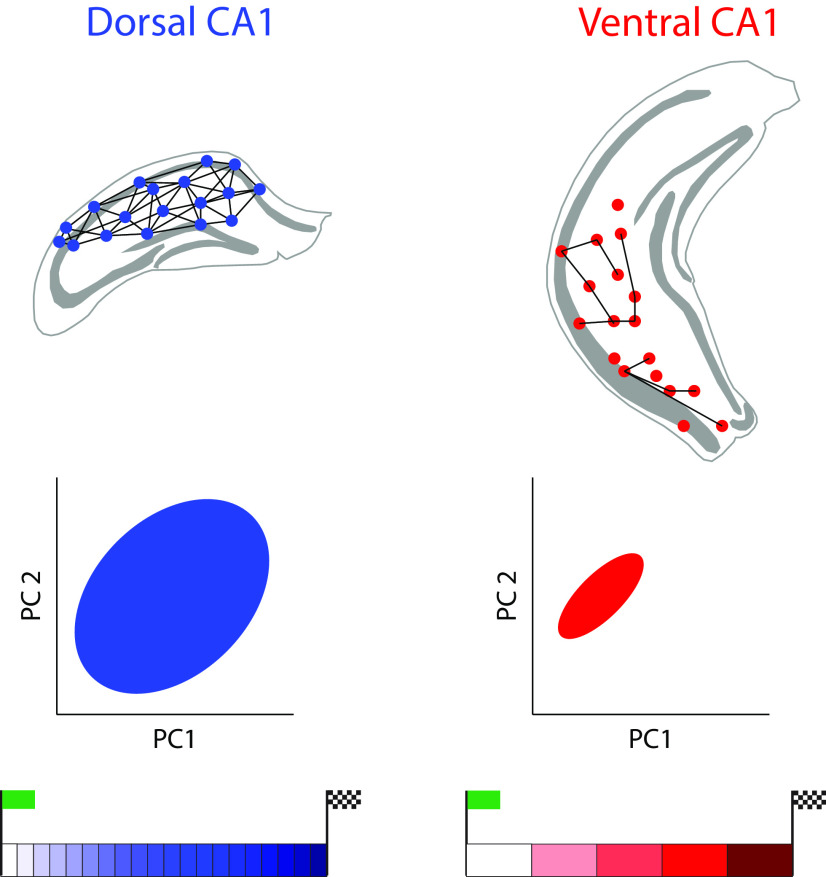

## Significance Statement

Recent studies have shown that spatial encoding in the hippocampus is not a simple function of individual place cell activities, but is rather sculpted by the collective activity of neuronal populations. However, the hippocampus is an anatomically and functionally heterogeneous structure, and it is not known how the features of this population code vary across the different hippocampal subfields. By performing recordings of large neuronal populations in dorsal and ventral CA1 as mice navigated a virtual environment, we showed how the functional architecture of the hippocampus underpins a divergent representation of space along its longitudinal axis, with ventral CA1 generating coarser and less precise spatial maps than dorsal CA1.

## Introduction

The hippocampus is a functionally diverse brain region, linked to an array of cognitive and emotional behaviors (Klüver and Bucy, 1937; Scoville and Milner, 1957). In rodents for example, this diversity is particularly pronounced along the dorsal-ventral axis of CA1, with dorsal CA1 implicated in episodic memory and spatial navigation in contrast to ventral CA1, which has been linked to anxiety and social cognition (Henke, 1990; Moser et al., 1995; Moser and Moser, 1998; Kjelstrup et al., 2002; Fanselow and Dong, 2010; Okuyama et al., 2016).

Functional diversity arises from differences at multiple scales in CA1, including differences in gene expression (Thompson et al., 2008; Cembrowski et al., 2016) to variations in intrinsic neuronal biophysical and morphologic properties (Dougherty et al., 2012, 2013; Malik et al., 2016) to diverse afferent and efferent anatomic connections (Swanson and Cowan, 1977; Cenquizca and Swanson, 2007; Meira et al., 2018; Padmanabhan et al., 2019). While these intrinsic and circuit-level differences contribute to the array of diverse behaviors linked to dorsal and ventral hippocampus, they also underlie differences of a single representation along the dorsal-ventral axis of the hippocampus. For example, place cells, neurons that preferentially fire when an animal visits a particular region of its environment (O’Keefe and Dostrovsky, 1971), are present throughout the dorsal-ventral axis of CA1. The place fields became progressive larger along this axis, with neurons in ventral CA1 providing less information about the animal’s position than those in dorsal CA1 (Jung et al., 1994; Ciocchi et al., 2015). As place cells are considered a critical component of the cognitive map, this suggests that the internal representation of space coarsens in ventral CA1 relative to dorsal CA1. However, multiple studies in dorsal hippocampus have demonstrated that the fidelity of spatial coding is not a simple function of place field size.

First, not all CA1 neurons are place cells. Numerous spatial navigation studies have demonstrated that the pool of place cells varies between environments, but that in any given environment, a subset of dorsal CA1 neurons do not show a place field (Wilson and McNaughton, 1993; Rich et al., 2014). Moreover, while most studies of place cells define a threshold of spatial information or sparsity (Skaggs et al., 1993), there is not a sharp boundary between place and non-place cells; dorsal CA1 neurons have variable amounts of spatial information and an animal’s position in space is likely encoded in combination with other variables, such as time, and reward (Gauthier and Tank, 2018; Haimerl et al., 2019; Stefanini et al., 2020). Activity of place cells is determined not only by the position of the animal, but also by correlations with other neurons, including non-place cells; these contributions from the broader population were able to partially explain the trial-to-trial variability in place cell responses (Meshulam et al., 2017). Neurons without clear place fields can thus be highly valuable for spatial coding, and place cells with high spatial information are not necessarily the only useful neurons in the population for decoding position (Stefanini et al., 2020).

Despite accumulating evidence that position information in the hippocampus is collectively encoded by groups of neurons, comparatively little is known about how this population activity varies across the dorsal-ventral axis of CA1 (Keinath et al., 2014). Does activity across the overall population compensate for the larger single-cell place fields in a way that maintains the precision of spatial representations across dorsal and ventral CA1? Alternatively, does the resolution of spatial coding in populations of neurons become coarser in ventral CA1, as it does for the place fields of individual neurons, suggesting divergent computational strategies for encoding place?

By recording from large neuronal populations from both regions as animals navigated a virtual track, we found a divergence in the population representation of space between dorsal and ventral CA1. Both at the single-neuron level and the population level, activity in dorsal CA1 was more informative about the animal’s position than that in ventral CA1. This increased spatial information was underpinned by more complex patterns of population activity in dorsal CA1 that increased the dimensionality of the neural code. To understand how these complex activity patterns may arise, we fit maximum entropy models to the data, which revealed that population-wide patterns could be predicted better using pairwise interactions between neurons in ventral CA1 as compared with dorsal CA1. Taken together, these results suggest that differences in the functional interactions between neurons across the longitudinal hippocampal axis result in differential coding strategies and divergent representations of space in dorsal and ventral CA1.

## Materials and Methods

### Mice

Four male C57BL6/J mice (RRID:IMSR_JAX:000664, The Jackson Laboratory) were included in this study. All mice were between 9 and 10 weeks of age at the time of recording. Mice were group-housed until headframe implantation, after which they were solo-housed. Mice were housed in transparent cages on a 12/12 h light/dark cycle. All recordings were performed in the light phase. Mice were healthy and were not used for any previous procedures. All procedures conformed to regulatory standards and were approved in advance by the Institutional Animal Care and Use Committee (IACUC) at the institution where these experiments were performed.

### Virtual reality setup

A virtual 1D 1.9-m track was created using the Virtual Reality MATLAB Engine (VirMEn) toolbox based on a previously published design (Meshulam et al., 2017; Gauthier and Tank, 2018). During the recording sessions, the position of the running wheel was transmitted to the computer by a two-bit rotational encoder attached to the axle. This information was used to update the position of the animal on the virtual track, which was also saved. The image was projected onto a curved board and occupied ∼180° of the visual field. When mice reached the end of the track, their virtual position was reset to the start of the track. The recording rig was enclosed in a box to minimize ambient light, odor, sound, and electromagnetic interference.

### Head fixing

Animals were anesthetized using a 1–2% isoflurane mixture and placed in a stereotactic surgical rig. The scalp was resected and the craniotomy sites for dorsal CA1 (coordinates relative to bregma: 2.5 mm caudal, 1.5 mm right) and ventral CA1 (coordinates relative to bregma: 3.15 mm caudal, 3.15 mm right). Subsequently, a metal ground pin and custom 3D-printed headframe was affixed to the skull using dental cement (Ortho-Jet Powder and Jet Liquid, Lang Dental Mfg. Co) and veterinary adhesive (Vetbond, The 3M Company). Postoperative analgesia was provided for 72 h with 0.1 mg/kg subcutaneous buprenorphine in accordance with approved protocols.

### Run training

During the 7-d period immediately following head frame implantation, animals were habituated to the virtual reality environment (Warner and Padmanabhan, 2020). Mice were head-fixed in the virtual environment and allowed to run for 1 h each day. Run behavior was recorded during this training period, but electrophysiological recordings were not performed.

### Electrophysiological recording

Animals were anesthetized using a 1–2% isoflurane mixture and a craniotomy was performed over the right dorsal and ventral CA1 hippocampal subfields. The craniotomy site was covered with a fast-curing silicone sealant (Kwik-Cast, World Precision Instruments) and mice were allowed to recover from anesthesia for 12–18 h in their home cages. The next day, mice were transferred to the running wheel and an open source microfabricated silicon electrode array (Du et al., 2011; Yang et al., 2020) was targeted to either dCA1 or vCA1. The 128DN probe configuration was used, which comprised 128 recording channels spread across four shanks spaced 150 μm apart. Extracellular voltage recordings were collected at 30 kHz in the 0.1- to 3500-Hz frequency band. Recordings were performed for at least 1 h at each recording site (for some animals, recordings were done at multiple regions of interest within dCA1 or vCA1). Afterwards, the electrode was removed and targeted to the other hippocampal subfield and the process was repeated (in half of the animals, dCA1 recordings were performed first, while in the other half, vCA1 recordings were performed first). The running behavior of the mouse and the position in the virtual track was also recorded simultaneously.

### Recording sites

Stereotactic coordinates for the recording sites were based on the Paxinos and Franklin atlas (Paxinos and Franklin, 2013): dorsal CA1 coordinates: 2.5 mm caudal, 1.5 mm right, 1 mm ventral; ventral CA1 coordinates: 3.15 mm caudal, 3.15 mm right, 4.25 mm ventral. While there is general consensus on the location of dorsal CA1, variation exists in the literature with respect to the boundaries of ventral CA1. This is often because different labs use different metrics (molecular, cellular, spatial, etc.) to delineate this region (Jung et al., 1994; Kjelstrup et al., 2008; Royer et al., 2010; Keinath et al., 2014; Ciocchi et al., 2015; Okuyama et al., 2016; Jimenez et al., 2018; Schmid et al., 2019). We targeted our ventral CA1 recording sites closer to the ventral boundary of CA1 associated with the stereotaxic coordinates above.

### Spike sorting

All computational analyses were performed in MATLAB R2019A (RRID: SCR_001622) on computers running Windows 10. The open source automated spike sorting toolbox Kilosort (RRID: SCR_016422; Pachitariu et al., 2016) was used to preprocess the electrophysiology recordings and identify the spike times of single units. The raw electrophysiology signal was high passed at 500 Hz, the median signal from all channels was subtracted from each channel, and correlated noise across channels was removed. subsequently, a set of template waveforms and spike times was generated and updated in an iterative manner to reconstruct the original data set. The waveforms and spike times at the end of this optimization process constituted putative single units. These were than manually curated using Phy (Rossant et al., 2016). Units were preserved, eliminated, or merged on the basis of their mean waveforms across multiple channels, amplitudes and inter-spike interval distributions. Units without clear refractory periods were excluded.

### Run behavior analysis

The two-bit wheel position information, recorded at 30 kHz, was converted to a velocity using a window of size 150 ms with 10-ms time steps. Intervals during which the running velocity was >5 cm/s were used for subsequent calculations (firing rates, correlations, entropy, spatial information, maximum entropy modeling, etc.). Only recording sessions during which the animal completed at least five full runs along the virtual track were included in the analyses.

### Single-cell spatial information

The vector of each animal’s position on the track was sorted into 111 bins, each of size ∼1.7 cm. For each unit, the mean firing rate in each bin was calculated and occupancy normalized. The resulting raw firing rate map was smoothed using a five-bin-width square wave and then normalized such that the maximum and minimum for each unit was 1 and 0, respectively. The spatial information of each unit was then calculated with the following formula (Skaggs et al., 1993):

$$I_{single-unit}=\underset{x}{\sum }-p_{x}\lambda_{x}\log_{2}\frac{\lambda }{\lambda_{x}}.$$

In this equation, $p_{x}$ denotes the probability that the animal is occupying spatial bin x, $\lambda_{x}$ denotes the firing rate in bin x, and λ denotes the mean firing rate across all bins. A complementary metric, sparsity, indicates the proportion of spatial bins over which a neuron is active (Skaggs et al., 1996):

$$S=\frac{{\left(\sum_{x}p_{x}\lambda_{x}\right)}^{2}}{\sum_{x}p_{x}{\left(\lambda_{x}\right)}^{2}}.$$

### Place field size and position

The center of a neuron’s place field was defined to the spatial bin in which the neuron’s firing rate was highest. The width of a place field was defined as the number of spatial bins around the place field center for which the neuron’s firing rate was at least 1.5 SDs above the mean. To ensure that the findings on place field size were not simply because of our choice of parameters, we performed three additional sets of analyses while varying these parameters.

First, we calculated place field size with only the subset of neurons with spatial information higher than expected by chance. We generated null distributions for spatial information using either a circular permutation method, in which the entire spike train of a given neuron was shifted by a random interval, or a random temporal shuffling of spikes. 1000 shuffled spike trains were generated for each neuron. Neurons that exceeded the 85th or the 95th percentiles were preserved for place field size calculations (Langston et al., 2010; Mao et al., 2017). Second, rather than smoothing the firing rate maps with a square wave, we recalculated place field sizes using Gaussian kernels of three different SDs: 0.1, 1, and 10 bins (Kjelstrup et al., 2008). Third, we determined the position of the place field center using the circular center of mass (Mehta et al., 1997; Meshulam et al., 2017), rather than the peak firing rate.

### Firing rate correlations and network representations

A continuous firing rate trace of each neuron was generated by calculating the number of spikes in each 50-ms interval of recording. The correlation coefficient between the firing rate traces of every pair of units in each animal was calculated. To convert the resulting correlation matrices to a graph, they were thresholded, such that the correlations that exceeded the threshold were preserved as an edge between the nodes representing that pair of neurons. The threshold was varied from 0 to 0.385 to cover the range of positive correlation strengths observed. For the network visualizations and pattern illustrations, the physical location of the units was approximated by the location of the electrode contact on which the largest mean spike waveform of each unit was detected.

### Principal components analysis (PCA)

The continuous firing rate traces used to calculate the correlations were also used to construct a covariance matrix. The eigenvectors of this matrix denoted the principal components, the axes along which the firing rates of the neuronal population showed the largest variance, and the eigenvalues denoted the variance along each principal component. For the scatter plots of low-dimensional population activity in Figure 5*A*,*B*, the firing rate traces were smoothed using a sliding Gaussian function with a SD of 1 s.

Because the number of neurons, and thus, the number of principal components, varied between recording sessions, the explained variance plots could not be directly compared across recording sessions. Instead, the explained variance was plotted against the fraction of the total principal components in each recording. The area under this curve was calculated directly. To approximate the fraction of principal components that explained 80% of the variance, a nonlinear least-squares curve was generated for each plot using the trust-region-reflective algorithm (Coleman and Li, 1996).

### Population entropy and information

The population spike rasters were placed into 10-ms bins and binarized such that the bin would be assigned a value of 0 if no spikes occurred in that 10-ms interval and a value of 1 if one or more spikes occurred. For these analyses, only recording sessions with >18 units were included. The combination of 0 and 1 from multiple units in the population in a given time bin constituted a pattern (de Ruyter van Steveninck et al., 1997; Schneidman et al., 2006; Chockanathan et al., 2020). The number of units used in this subsample was varied from 4 units (in which case there were 2^4^ = 16 possible binary patterns) to 24 units (2^24^ = 16777216 possible patterns). The total entropy of the population was calculated from the equation, in which $p_{k}$ denotes the probability of the k^th^ pattern:

$$H_{total}=\underset{k}{\sum }-p_{k}\log_{2}p_{k}.$$

The total entropy describes the overall diversity of observed population patterns. However, the distribution of population patterns varied as a function of the animal’s position on the track. These conditional pattern probabilities $\left(p_{k}|x\right)$ were used to calculate the entropy as a function of animal’s position (the spatial bins used here were the same as those used to calculate the single-cell spatial information):

$$H_{x}=\underset{k}{\sum }(-p_{k}|x)\log_{2}(p_{k}|x).$$

The average of all of these conditional entropies, weighted by the occupancy probability for each spatial bin, constitutes the noise entropy:

$$H_{noise}=\underset{x}{\sum }p_{x}H_{x}.$$

The spatial information of the population was calculated by the difference between the total entropy and the noise entropy:

$$I_{population}=H_{total}-H_{noise}.$$

### Maximum entropy modeling

Maximum entropy models were fit to the data using the maxent_toolbox (Maoz and Schneidman, 2017). First, spike trains were binarized using 10-ms non-overlapping bins, as in the entropy analysis. Second, subpopulations of *n* = 4–18 units were randomly generated. Only recording sessions with >18 units were included in these analyses. Third, for every neuron, a local field term *h_i_* was calculated. This term denotes the activity of that unit. Fourth, for every pair of units in the subsample, a pairwise interaction term *J_ij_* was calculated. This term describes the level of functional coupling between pairs of units. Subsequently, the probabilities of population patterns were predicted using only these *h_i_* and *J_ij_* terms:

$$P_{predicted}\left(\sigma_{1},\sigma_{2}...\sigma_{n}\right)=\frac{1}{Z}e^{\underset{i}{\sum }h_{i}\sigma_{i}+\frac{1}{2}\underset{i\neq j}{\sum }J_{ij}\sigma_{i}\sigma_{j}}.$$

In this equation, $\sigma_{i}$ indicates the binary state of the i^th^ unit and Z indicates the partition function used to normalize the probability distribution. Thus, the model attempts to predict the activity patterns of many-neuron populations without taking into account any higher-order interactions beyond pairwise couplings. The predicted pattern probabilities were then compared with the corresponding empirical probabilities and the prediction error was quantified using the Kullback–Liebler divergence (KLD).

### Quantification and statistical analysis

For all hypothesis testing, two-sample Wilcoxon rank sum tests were performed, as the data were not assumed to be normally distributed. The α value was set at 0.05, and Bonferroni corrections were applied when multiple comparisons were performed. Details of statistical parameters (sample sizes used for statistical comparison, meaning of error bars, *p* values) are reported in the figure legends or in the relevant section of the main text.

## Results

### Head-fixed dorsal and ventral CA1 population recordings in virtual reality

**Figure 1.**
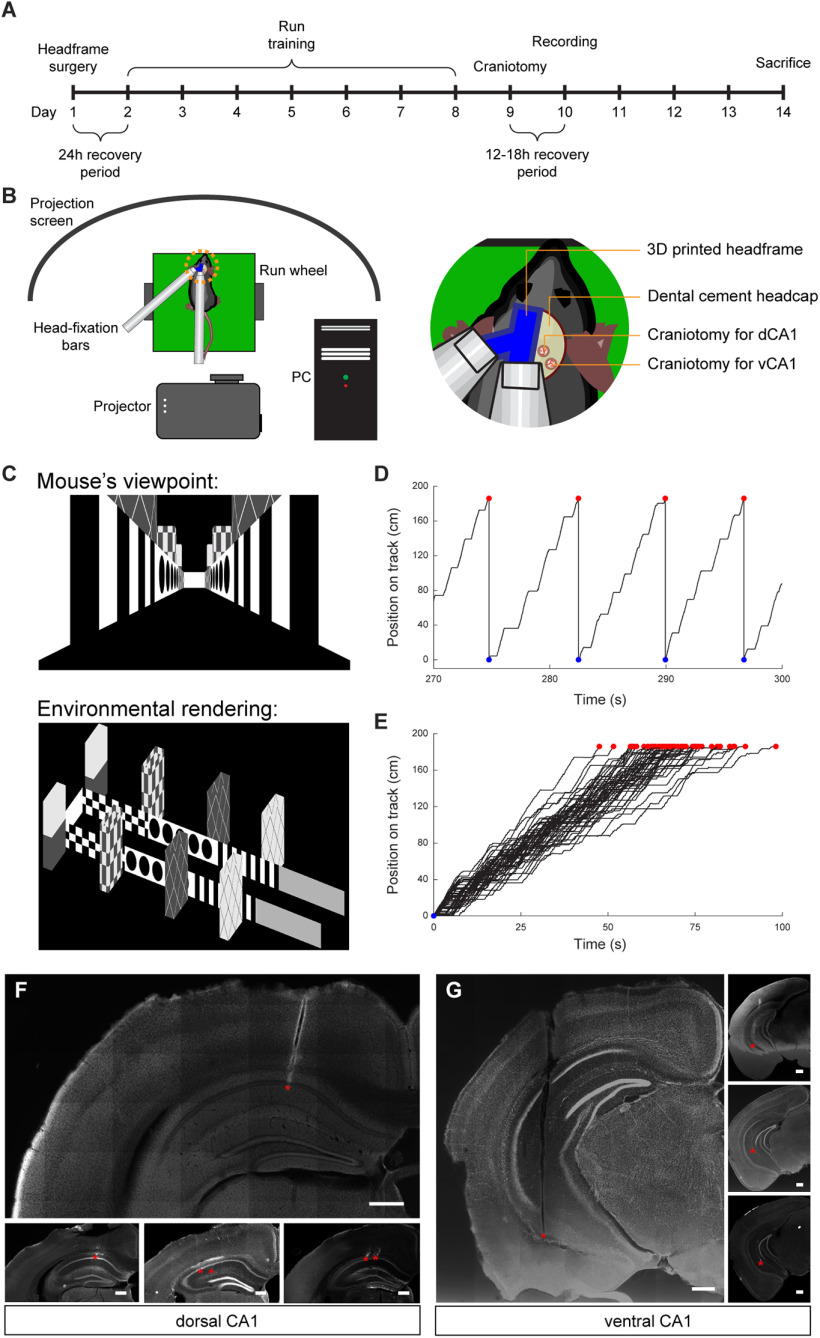
Head-fixed recordings of neuronal populations in virtual reality. ***A***, Timeline of experimental procedures. ***B***, Schematic of recording setup with virtual environment. ***C***, top, Sample frame of virtual environment, shown from the perspective of the mouse. Bottom, Schematic of 1.9-m virtual track. ***D***, Sample of animal trajectory through virtual track. When mice reached end of track (red circles), they were transported to the start of the track (blue circles). ***E***, Superimposed trajectories of all laps completed in one representative recording session. ***F***, Histologic section showing the placement of recording electrodes in dorsal CA1. Red asterisks denote the tips of the electrode tracks. Each panel shows a section from a different animal. Scale bars: 400 μm. ***G***, Histologic section showing the placement of recording electrodes in ventral CA1. Red asterisks denote the tips of the electrode tracks. Each panel shows a section from a different animal. Scale bars: 400 μm.

To compare the features of neuronal population activity across the dorsal-ventral axis of CA1 hippocampus, we performed electrophysiological extracellular recordings in awake head-fixed mice on a running wheel in a virtual environment. Four male C57BL6/J mice of 9–10 weeks of age were head-fixed and trained to run through a virtual 1D virtual track (Fig. 1*A–C*; for details, see Materials and Methods; Gauthier and Tank, 2018). All locomotion was self-motivated; the wheel was non-motorized and no reward was provided. Over the 7-d period, mice learned to complete multiple runs on the track (mean ± standard deviation: 37 ± 22 laps per recording session; Fig. 1*D*,*E*). The mice were then anesthetized and a craniotomy was performed over dorsal and ventral CA1. After a 12- to 18-h recovery period, the animals were again placed on the run-wheel. A high-density 128-channel silicon electrode array was lowered to either dorsal or ventral CA1 (Fig. 1*F*,*G*). There were no significant behavioral differences in running, including number of laps run, fraction of time spent running, and mean running velocity, between dorsal CA1 and ventral CA1 recording sessions (Extended Data Fig. 1-1).

10.1523/ENEURO.0211-21.2021.f1-1 Extended Data Figure 1-1 No significant differences in running behavior between recording sessions in dorsal and ventral CA1. ***A***, No difference in number of laps run between dorsal and ventral CA1 recording sessions (mean ± std: dCA1 = 38.43 ± 26.07 laps, vCA1 = 34.67 ± 18.95 laps, *p *=* *0.98, two-sided Wilcoxon rank-sum test, *n*_dCA1_ = 7 recording sessions, *n*_vCA1_ = 6 recording sessions). Each point denotes a recording session. ***B***, No difference in fraction of time spent running between dorsal and ventral CA1 recording sessions (mean ± std: dCA1 = 0.14 ± 0.06, vCA1 = 0.16 ± 0.11 laps, *p *=* *0.95, two-sided Wilcoxon rank-sum test, *n*_dCA1_ = 7 recording sessions, *n*_vCA1_ = 6 recording sessions). Each point denotes a recording session. ***C***, No difference in mean running speed between dorsal and ventral CA1 recording sessions (mean ± std: dCA1 = 8.71 ± 1.17 cm/s, vCA1 = 8.72 ± 1.19 laps, *p *=* *0.95, two-sided Wilcoxon rank-sum test, *n*_dCA1_ = 7 recording sessions, *n*_vCA1_ = 6 recording sessions). Each point denotes a recording session. Download Figure 1-1, EPS file.

**Figure 2.**
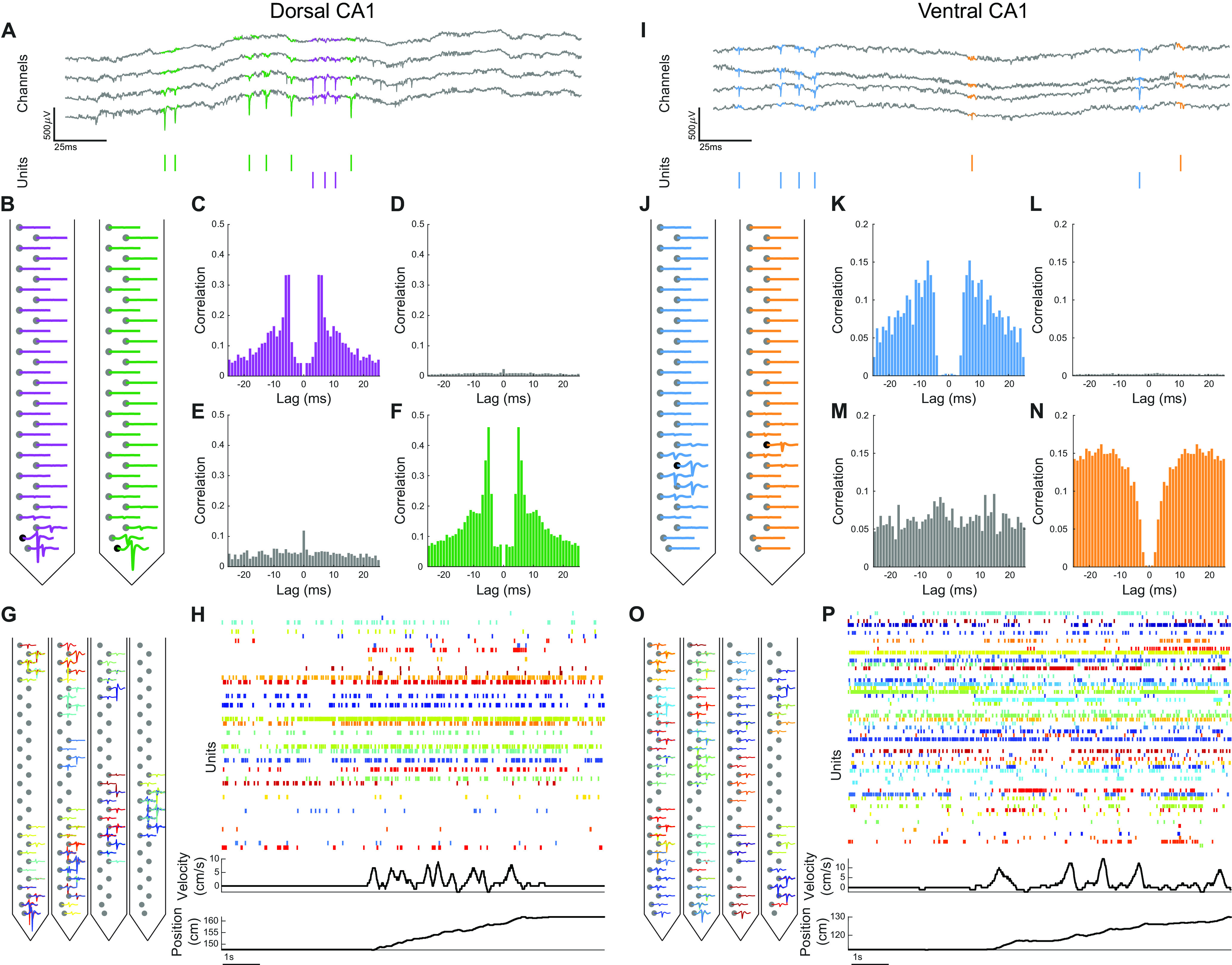
Spike sorting to isolate single-unit activity. ***A***, ***I***, top, Sample trace of wide-band (0.1–3500 Hz) electrophysiological recording in dorsal CA1 (***A***) and ventral CA1 (***I***). Spike waveforms from different units are highlighted in different colors. Bottom, Raster plot denoting spike times for each unit. ***B***, ***J***, Mean waveform of each unit in ***A***, ***I*** across all 32 channels on a single shank of the electrode array. ***C***, ***F***, ***K***, ***N***, Autocorrelograms for units in ***A***, ***I***, showing clear refractory periods. ***D***, ***E***, ***L***, ***M***, Cross-correlograms for units in ***A***, ***I***, showing no refractory period. ***G***, ***O***, Mean waveform of all units from a single dorsal CA1 (***G***) or ventral CA1 (***O***) recording session with each color denoting a single unit. For each unit, waveforms are shown on the channel with the largest amplitude spike as well as three neighboring channels. ***H***, ***P***, top, Sample of dorsal CA1 (***H***) and ventral CA1 (***P***) population activity. The color of the raster plot row corresponds to the unit waveforms in ***G***, ***O***. Middle, Simultaneous running velocity of animal. Bottom, Simultaneous position of animal on virtual track.

For both dorsal and ventral CA1, individual action potentials, or spikes, were observed in the broadband voltage traces (Fig. 2*A*,*I*). Template matching with Kilosort (Pachitariu et al., 2016) was used to cluster the spike waveforms and identify putative units across multiple neighboring channels. The resulting units were then curated using Phy (Rossant et al., 2016), only those with asymmetric, spike-like waveforms (Fig. 2*B*,*J*), as well as a clear refractory period in their autocorrelograms (Fig. 2*C*,*F*,*K*,*N*) were included in analysis. Units were merged based on the similarity of their waveforms and the features of their cross-correlograms (Fig. 2*D*,*E*,*L*,*M*) were merged. This process yielded between 42 and 80 units per recording in dorsal CA1 and between 43 and 62 units per recording in ventral CA1 (Fig. 2*G*,*O*). There was no significant difference in the number of units recorded during the dorsal and ventral CA1 recording sessions (Extended Data Fig. 2-1). The resulting neuronal populations revealed complex patterns of activity in both regions as the animals navigated the virtual space (Fig. 2*H*,*P*).

10.1523/ENEURO.0211-21.2021.f2-1 Extended Data Figure 2-1 No significant differences in number of units recorded in dorsal and ventral CA1 recording sessions. No difference in number of units recorded in the two regions (mean ± std: dCA1 = 47.86 ± 22.25 units, vCA1 = 38.00 ± 18.41 laps, *p *=* *0.42, two-sided Wilcoxon rank-sum test, *n*_dCA1_ = 7 recording sessions, *n*_vCA1_ = 6 recording sessions). Each point denotes a recording session. Download Figure 2-1, EPS file.

### Coarsening of single-neuron spatial representations along dorsal-ventral axis of CA1

**Figure 3.**
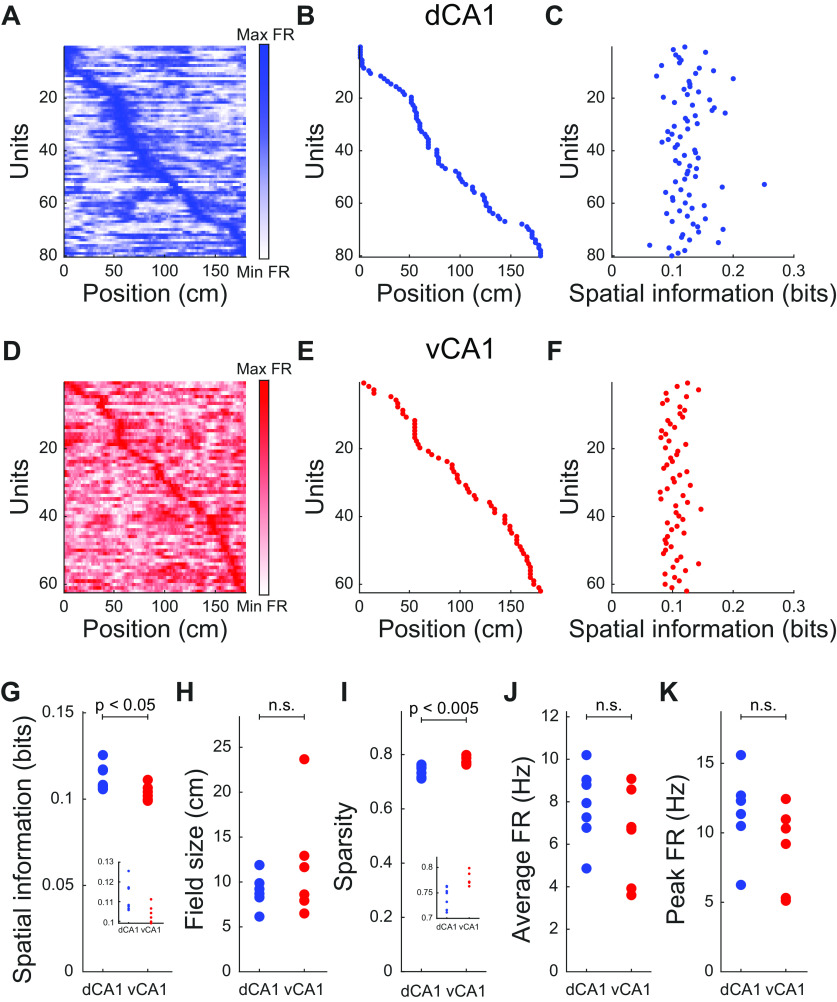
Ventral CA1 neurons have lower spatial information than dorsal CA1 neurons. ***A***, ***D***, Map of single-neuron activity as a function of animal position in virtual track for a representative recording session in dorsal CA1 (***A***) and ventral CA1 (***D***). Each row denotes a single unit. ***B***, ***E***, Location in virtual track of peak firing rate for each unit in ***A***, ***D***. ***C***, ***F***, Spatial information of each unit in ***A***, ***D***. ***G***, Dorsal CA1 neurons had larger spatial information than ventral CA1 neurons (mean ± std: dCA1 = 0.113 ± 0.007 bits, vCA1 = 0.104 ± 0.005 bits, *p *=* *0.04, two-sided Wilcoxon rank-sum test, *n*_dCA1_ = 7 recording sessions, *n*_vCA1_ = 6 recording sessions). Inset shows magnification. Each point denotes a recording session. ***H***, Place field sizes were not significantly different for dorsal and ventral CA1 neurons (mean ± std: dCA1 = 9.4 ± 2.0 cm, vCA1 = 11.9 ± 6.3 cm, *p *=* *0.84, two-sided Wilcoxon rank-sum test, *n*_dCA1_ = 7 recording sessions, *n*_vCA1_ = 6 recording sessions). Each point denotes a recording session. ***I***, Dorsal CA1 neurons had sparser activity than ventral CA1 neurons (mean ± std: dCA1 = 0.74 ± 0.02, vCA1 = 0.78 ± 0.01, *p *=* *0.002, two-sided Wilcoxon rank-sum test, *n*_dCA1_ = 7 recording sessions, *n*_vCA1_ = 6 recording sessions). Inset shows magnification. Each point denotes a recording session. ***J***, Average firing rates were not significantly different for dorsal and ventral CA1 neurons (mean ± std: dCA1 = 7.84 ± 1.74 Hz, vCA1 = 6.45 ± 2.29 Hz, *p *=* *0.30, two-sided Wilcoxon rank-sum test, *n*_dCA1_ = 7 recording sessions, *n*_vCA1_ = 6 recording sessions). Each point denotes a recording session. ***K***, Peak firing rates were not significantly different for dorsal and ventral CA1 neurons (mean ± std: dCA1 = 11.57 ± 2.83 Hz, vCA1 = 8.89 ± 3.04 Hz, *p *=* *0.10, two-sided Wilcoxon rank-sum test, *n*_dCA1_ = 7 recording sessions, *n*_vCA1_ = 6 recording sessions). Each point denotes a recording session.

Previous studies using rodents in an open field have shown that the neuronal representation of space varies along the dorsal-ventral axis of the hippocampus, with ventral CA1 neurons showing more diffuse place fields and lower spatial information than dorsal CA1 neurons (Jung et al., 1994; Kjelstrup et al., 2008; Keinath et al., 2014; Ciocchi et al., 2015). To test whether this held true in a virtual environment with head-fixed animals, we first visualized the activity of each neuron as a function of the animal’s position along the virtual track (Fig. 3*A*,*D*). In both dorsal and ventral CA1 neurons, we observed place fields that tiled the length of the track (Fig. 3*B*,*E*).

We quantified these activity patterns by calculating the spatial information (Fig. 3*C*,*F*; Skaggs et al., 1993), a measure of how the neuron’s firing rate predicted the animal’s location. Ventral CA1 neurons had a lower information content than dorsal CA1 neurons (Fig. 3*G*), consistent with previous studies on freely behaving animals in open-field environments (Jung et al., 1994). Surprisingly, we found that the decreased information content in ventral CA1 neurons was not because of larger place fields, as the place field size was not significantly different between the two regions (Fig. 3*H*). Instead, this reduction in spatial information in ventral CA1 was because of a higher level of spontaneous firing at track positions not associated with the place field. This was quantified by the sparsity metric (Skaggs et al., 1996), which showed that ventral CA1 neurons were active over a larger fraction of the track than dorsal CA1 neurons (Fig. 3*I*). Importantly, the difference in spatial information between the two regions could not be explained by differences in either the average firing rates or the peak firing rates, which were not significantly different between ventral and dorsal CA1 neurons (Fig. 3*J*,*K*). Additionally, the relationship between mean firing rates and spatial information was not significantly different for the two regions (Extended Data Fig. 3-1).

10.1523/ENEURO.0211-21.2021.f3-1 Extended Data Figure 3-1 Association between spatial information, firing rate, and place field location is preserved for dorsal and ventral CA1 neurons. ***A***, Scatter plot of spatial information of each neuron against the location of its place field. There is no correlation between these variables in dorsal CA1 (*r* = –0.0044, *p *=* *0.9366, Fisher transformation with two-sided Z-test, *n* = 335 neurons) nor in ventral CA1 (*r *=* *0.0047, *p *=* *0.9467, Fisher transformation with two-sided Z-test, *n* = 228 neurons). Moreover, this correlation coefficient is not significantly different between dorsal and ventral CA1 (*r_dCA1_* = –0.0044, *r_vCA1_* = 0.0047, *p *=* *0.9124, Fisher transformation with two-sided Z-test, *n*_dCA1_ = 335 neurons, *n*_vCA1_ = 228 neurons). Each point denotes a single neuron, the black line denotes the least-squares regression, and the shaded area denotes the 95% confidence interval of the regression line. The text box shows *r*, the Pearson correlation coefficient, and the corresponding *p* value. ***B***, Scatter plot of spatial information of each neuron against its mean firing rate. These variables are correlated in both dorsal CA1 (*r* = –0.1482, *p *=* *0.0066, Fisher transformation with two-sided Z-test, *n* = 335 neurons) and ventral CA1 (*r* = –0.1302, *p *=* *0.0496, Fisher transformation with two-sided Z-test, *n* = 228 neurons). However, this correlation coefficient is not significantly different between dorsal and ventral CA1 (*r_dCA1_* = –0.1482, *r_vCA1_* = –0.1302, *p *=* *0.8336, Fisher transformation with two-sided Z-test, *n*_dCA1_ = 335 neurons, *n*_vCA1_ = 228 neurons). Each point denotes a single neuron, the black line denotes the least-squares regression, and the shaded area denotes the 95% confidence interval of the regression line. The text box shows *r*, the Pearson correlation coefficient, and the corresponding *p* value. Download Figure 3-1, EPS file.

This finding of similar place field sizes in dorsal and ventral CA1 was surprising, and we wanted to ensure that it was not simply because of our choice of analysis parameters. Thus, we recalculated place field sizes while varying our analyses methods. First, we restricted our analysis to only those neurons with high spatial information (Langston et al., 2010; Mao et al., 2017; Extended Data Fig. 3-2). Second, we used a Gaussian kernel rather than a square wave to smooth the firing rate maps (Kjelstrup et al., 2008; Extended Data Fig. 3-3). Third, we determined place field centers using a circular center of mass (Mehta et al., 1997; Meshulam et al., 2017; Extended Data Fig. 3-4). Finally, we compared place field size and spatial information between the two regions at the single-neuron level (Extended Data Fig. 3-5). In all of these cases, the original result held true; place field size was not significantly different between dorsal and ventral CA1. We also mapped the location of place fields along the virtual track and found that there was no significant enrichment or depletion of place fields at any location (Extended Data Fig. 3-6).

10.1523/ENEURO.0211-21.2021.f3-2 Extended Data Figure 3-2 Analysis of place field sizes when only neurons with high spatial information are included. ***A***, The spike train from each neuron was circularly shifted by a random interval 1000 times to generate a null distribution. Only neurons with spatial information that exceeded the 95th percentile of this shuffled distribution were included in this analysis. There was no difference in place field sizes between dorsal and ventral CA1 neurons (mean ± std: dCA1 = 6.982 ± 3.254 cm, vCA1 = 12.149 ± 9.762 cm, *p *=* *0.70, two-sided Wilcoxon rank-sum test, *n*_dCA1_ = 3 recording sessions, *n*_vCA1_ = 3 recording sessions). Each point denotes a recording session. ***B***, The spike train from each neuron was circularly shifted by a random interval 1000 times to generate a null distribution. Only neurons with spatial information that exceeded the 85th percentile of this shuffled distribution were included in this analysis. There was no difference in place field sizes between dorsal and ventral CA1 neurons (mean ± std: dCA1 = 10.700 ± 3.526 cm, vCA1 = 25.065 ± 33.703 cm, *p *=* *0.90, two-sided Wilcoxon rank-sum test, *n*_dCA1_ = 5 recording sessions, *n*_vCA1_ = 4 recording sessions). Each point denotes a recording session. ***C***, The spike train from each neuron was temporally shuffled 1000 times to generate a null distribution. Only neurons with spatial information that exceeded the 95th percentile of this shuffled distribution were included in this analysis. There was no difference in place field sizes between dorsal and ventral CA1 neurons (mean ± std: dCA1 = 8.303 ± 1.693 cm, vCA1 = 13.784 ± 13.601 cm, *p *=* *1, two-sided Wilcoxon rank-sum test, *n*_dCA1_ = 7 recording sessions, *n*_vCA1_ = 5 recording sessions). Each point denotes a recording session. ***D***, Neurons with high spatial information were identified using the same circular permutation shuffling procedure as ***A***, but each neuron is considered as a separate data point. There was no difference in place field sizes between dorsal and ventral CA1 neurons (mean ± std: dCA1 = 7.261 ± 3.021 cm, vCA1 = 14.336 ± 23.803 cm, *p *=* *0.91, two-sided Wilcoxon rank-sum test, *n*_dCA1_ = 9 neurons, *n*_vCA1_ = 9 neurons). ***E***, Neurons with high spatial information were identified using the same circular permutation shuffling procedure as ***B***, but each neuron is considered as a separate data point. There was no difference in place field sizes between dorsal and ventral CA1 neurons (mean ± std: dCA1 = 11.214 ± 13.252 cm, vCA1 = 11.544 ± 18.834 cm, *p *=* *0.45, two-sided Wilcoxon rank-sum test, *n*_dCA1_ = 26 neurons, *n*_vCA1_ = 27 neurons). ***F***, Neurons with high spatial information were identified using the same temporal shuffling procedure as ***C***, but each neuron is considered as a separate data point. There was no difference in place field sizes between dorsal and ventral CA1 neurons (mean ± std: dCA1 = 8.788 ± 4.647 cm, vCA1 = 13.091 ± 21.402 cm, *p *=* *0.13, two-sided Wilcoxon rank-sum test, *n*_dCA1_ = 45 neurons, *n*_vCA1_ = 32 neurons). Download Figure 3-2, EPS file.

10.1523/ENEURO.0211-21.2021.f3-3 Extended Data Figure 3-3 Analysis of place field sizes when firing rate maps are smoothed with a gaussian kernel. ***A***, There was no difference in place field sizes between dorsal and ventral CA1 neurons when the firing rate maps were smoothed using a Gaussian kernel with a SD of 0.1 bins (mean ± std: dCA1 = 14.534 ± 3.492 cm, vCA1 = 17.242 ± 2.616 cm, *p *=* *0.14, two-sided Wilcoxon rank-sum test, *n*_dCA1_ = 7 recording sessions, *n*_vCA1_ = 6 recording sessions). Each point denotes a recording session. ***B***, There was no difference in place field sizes between dorsal and ventral CA1 neurons when the firing rate maps were smoothed using a Gaussian kernel with a SD of 1 bin (mean ± std: dCA1 = 8.520 ± 2.585 cm, vCA1 = 10.880 ± 2.953 cm, *p *=* *0.14, two-sided Wilcoxon rank-sum test, *n*_dCA1_ = 7 recording sessions, *n*_vCA1_ = 6 recording sessions). Each point denotes a recording session. ***C***, There was no difference in place field sizes between dorsal and ventral CA1 neurons when the firing rate maps were smoothed using a Gaussian kernel with a SD of 10 bins (mean ± std: dCA1 = 10.657 ± 1.449 cm, vCA1 = 9.084 ± 1.337 cm, *p *=* *0.10, two-sided Wilcoxon rank-sum test, *n*_dCA1_ = 7 recording sessions, *n*_vCA1_ = 6 recording sessions). Each point denotes a recording session. Download Figure 3-3, EPS file.

**Figure 4.**
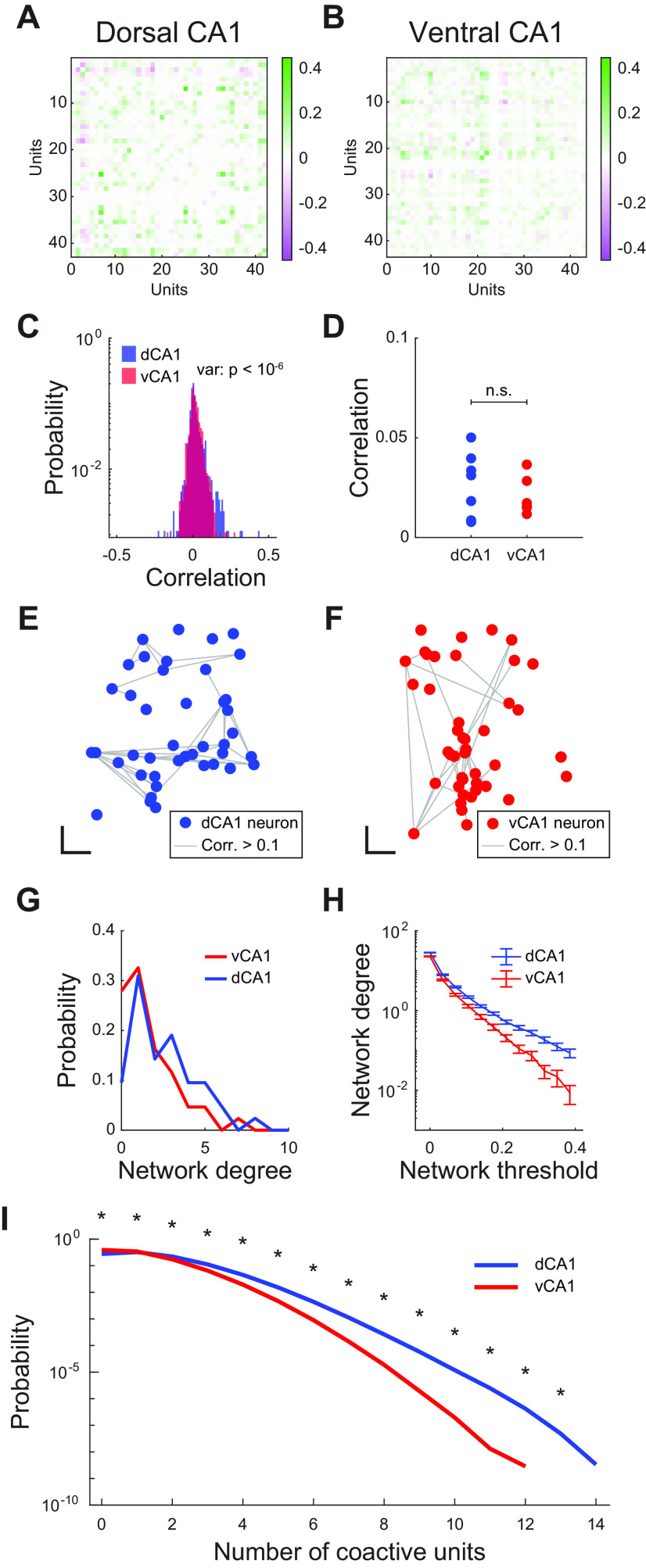
Preserved correlations, but weaker network structure in ventral CA1. ***A***, ***B***, Matrix of Pearson correlation coefficients for every pair of units in a representative recording session in dorsal CA1 (***A***) and ventral CA1 (***B***). Each grid cell denotes the correlation coefficient for a single pair of units. ***C***, Distribution of correlation coefficients for the two example sessions in ***A***, ***B***. There was no significant difference in the mean correlation between dorsal and ventral CA1 (mean ± std: dCA1 = 0.018 ± 0.049, vCA1 = 0.018 ± 0.038, *p *=* *0.06, two-sided Wilcoxon rank-sum test, *n*_dCA1_ = 861 pairs of units, *n*_vCA1_ = 903 pairs of units). However, the variance of the correlation distribution was larger in dorsal CA1 than in ventral CA1 (variance: dCA1 = 2.4 × 10^−3^; vCA1 = 1.4 × 10^−3^, *p *<* *10^−6^, two-sided *F* test for variance, *n*_dCA1_ = 861 pairs of units, *n*_vCA1_ = 903 pairs of units). ***D***, Average correlation coefficients were not significantly different for dorsal and ventral CA1 neurons (mean ± std: dCA1 = 0.027 ± 0.016, vCA1 = 0.021 ± 0.010, *p *=* *0.53, two-sided Wilcoxon rank-sum test, *n*_dCA1_ = 7 recording sessions, *n*_vCA1_ = 6 recording sessions). Each point denotes a recording session. ***E***, ***F***, Network representation of correlations for a representative dorsal CA1 (***E***) and ventral CA1 (***F***) recording session. Points denote the approximate physical position of individual neurons (jitter added to minimize overlapping neurons) and gray lines denote suprathreshold correlations between pairs of neurons. Scale bars: 100 μm (horizontal and vertical directions). ***G***, Distribution of network degree (number of incident edges) for the representative example networks in ***E***, ***F***. ***H***, Mean degree of networks in dorsal CA1 was higher than that of ventral CA1 networks across a range of network thresholds linearly increasing from 0 to 0.385 (for each threshold: *p *<* *0.005, two-sided Wilcoxon rank-sum test, *n*_dCA1_ = 335 units, *n*_vCA1_ = 228 units). Error bars denote SEM. ***I***, Mean probability of population patterns grouped by number of coactive units, generated using 1000 18-unit subsamples in each recording session. Asterisks denote significant difference between dorsal and ventral CA1 probabilities (for each pattern type: *p *<* *10^−6^, two-sided Wilcoxon rank-sum test, *n*_dCA1_ = 6000 samples from 6 recording sessions in 3 animals, *n*_vCA1_ = 4000 samples from 4 recording sessions in 2 animals).

10.1523/ENEURO.0211-21.2021.f3-4 Extended Data Figure 3-4 Analysis of place field sizes using center of mass to define place field center. There was no significant difference in place field size between dorsal and ventral CA1 when the center of the place field was defined using the circular center of mass (mean ± std: dCA1 = 8.591 ± 2.028 cm, vCA1 = 6.764 ± 2.031 cm, *p *=* *0.11, two-sided Wilcoxon rank-sum test, *n*_dCA1_ = 7 recording sessions, *n*_vCA1_ = 5 recording sessions). Download Figure 3-4, EPS file.

**Figure 5.**
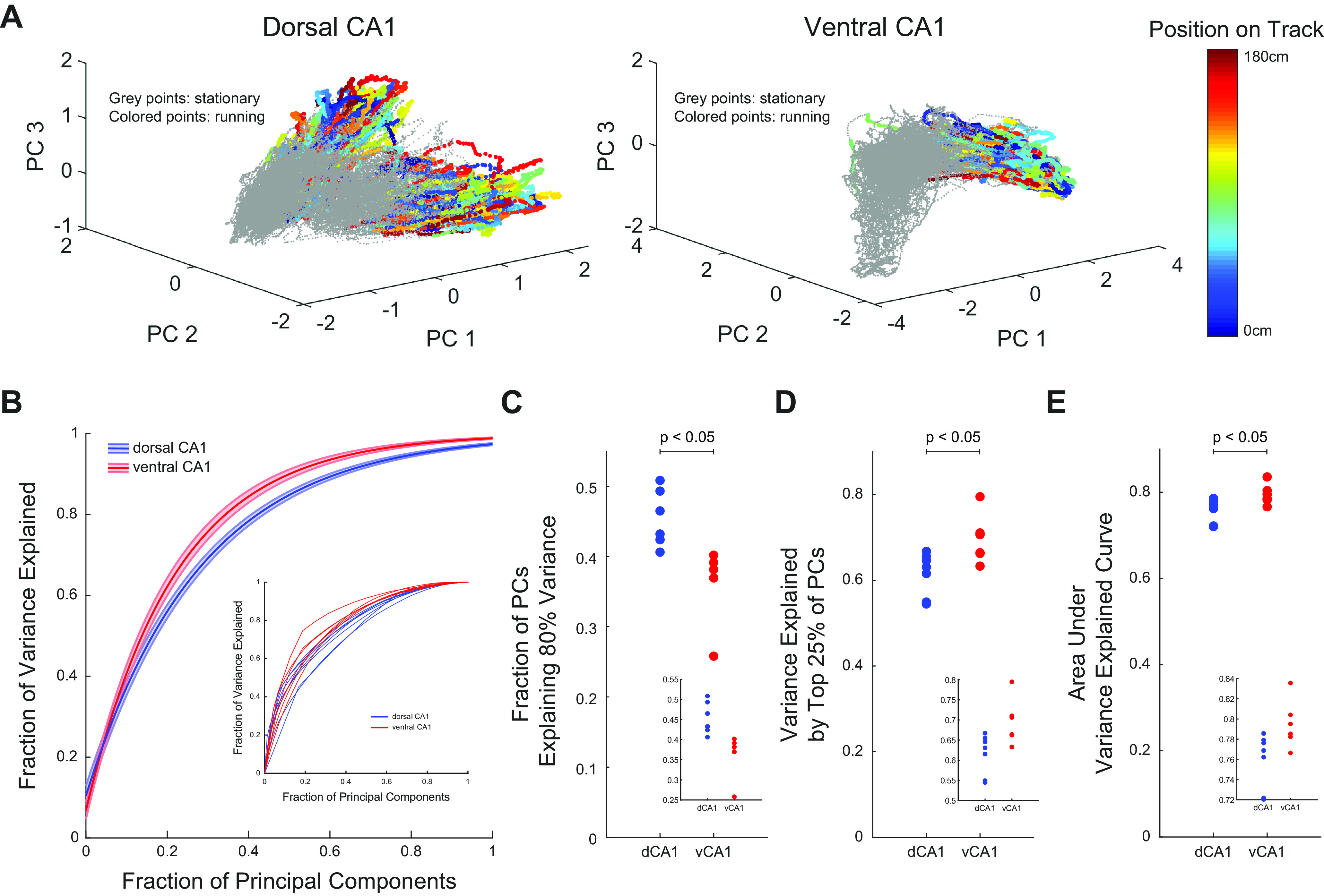
Dimensionality of population activity is reduced in ventral CA1. ***A***, Projections of population firing rates onto low-dimensional space defined by first three principal components for a representative dorsal CA1 (left) and ventral CA1 (right) recording session. ***B***, Cumulative explained variance as a function of fraction of total principal components ordered by their corresponding eigenvalues. Shaded regions denote SEM. Inset, Explained variance curves for single recording sessions. ***C***, A larger fraction of principal components was required to explain 80% of the variance in dorsal CA1 population than in ventral CA1 populations (mean ± std: dCA1 = 0.422 ± 0.040, vCA1 = 0.344 ± 0.046, *p *=* *0.035, two-sided Wilcoxon rank-sum test, *n*_dCA1_ = 7 recording sessions, *n*_vCA1_ = 6 recording sessions). Inset shows magnification. Each point denotes a recording session. ***D***, The first 25% of principal components explain a larger fraction of the variance in ventral CA1 than in dorsal CA1 (mean ± std: dCA1 = 0.632 ± 0.040, vCA1 = 0.697 ± 0.050, *p *=* *0.035, two-sided Wilcoxon rank-sum test, *n*_dCA1_ = 7 recording sessions, *n*_vCA1_ = 6 recording sessions). Inset shows magnification. Each point denotes a recording session. ***E***, The area under the variance explained curve was larger for ventral CA1 populations than for dorsal CA1 populations (mean ± std: dCA1 = 0.759 ± 0.027, vCA1 = 0.795 ± 0.024, *p *=* *0.035, two-sided Wilcoxon rank-sum test, *n*_dCA1_ = 7 recording sessions, *n*_vCA1_ = 6 recording sessions). Inset shows magnification. Each point denotes a recording session.

10.1523/ENEURO.0211-21.2021.f3-5 Extended Data Figure 3-5 Analysis of place field sizes and spatial information at the single-cell level. ***A***, There was no significant difference in place field size between dorsal and ventral CA1 when each neuron was considered an independent data point (mean ± std: dCA1 = 9.942 ± 10.935 cm, vCA1 = 10.114 ± 12.910 cm, *p *=* *0.05, two-sided Wilcoxon rank-sum test, *n*_dCA1_ = 329 neurons, *n*_vCA1_ = 196 neurons). ***B***, Single-neuron spatial information was higher in dorsal CA1 than ventral CA1 when each neuron was considered an independent data point (mean ± std: dCA1 = 0.113 ± 0.025 bits, vCA1 = 0.1056 ± 0.020 bits, *p *<* *10^−4^, two-sided Wilcoxon rank-sum test, *n*_dCA1_ = 329 neurons, *n*_vCA1_ = 196 neurons). Download Figure 3-5, EPS file.

**Figure 6.**
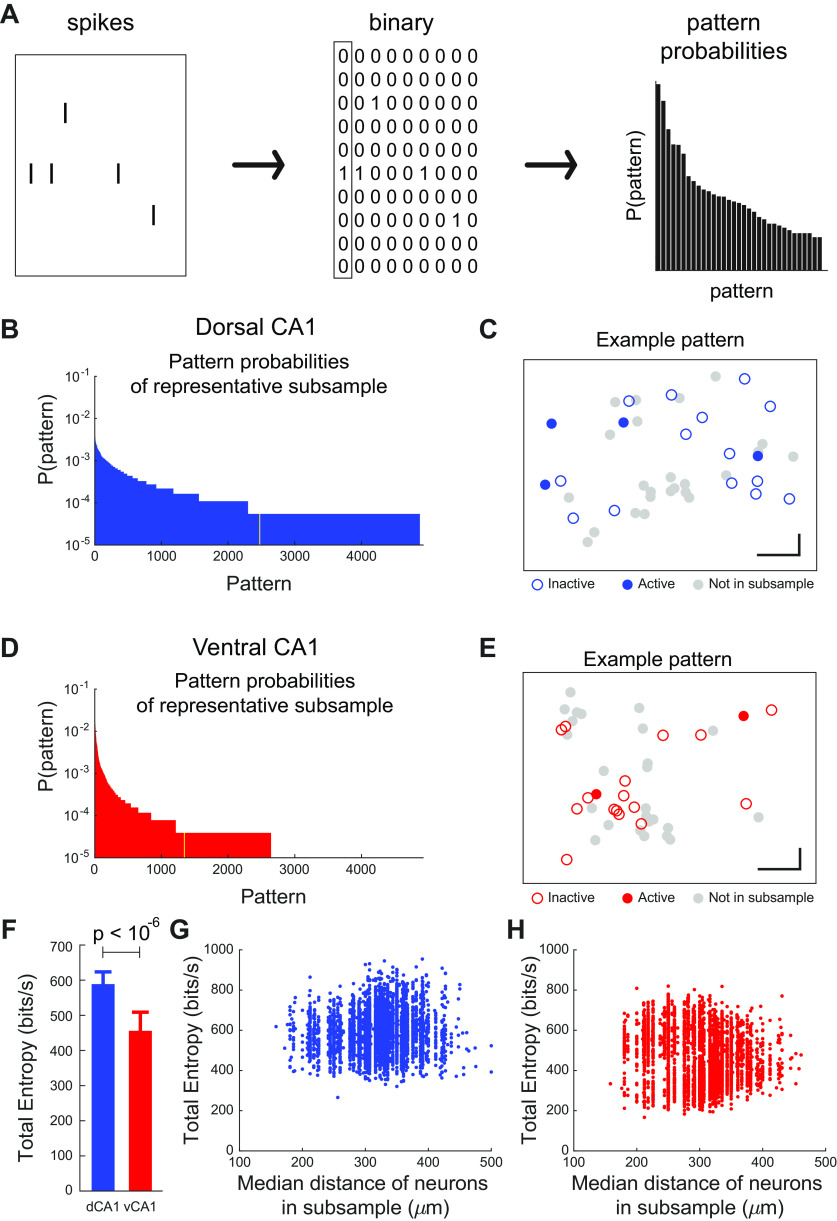
More diverse and complex population patterns in dorsal CA1. ***A***, Schematic for calculating pattern probabilities. Left, The spike rasters for each unit were binned using 10-ms non-overlapping windows and assigned a value of 0 if no spikes were present and a value of 1 if one or more spikes were present. Center, The combination of these binary states across all neurons in the subsample in a given window was denoted as a pattern. Right, Histogram of pattern probabilities. ***B***, ***D***, Distribution of pattern probabilities for a single 18-unit subsample of a dorsal CA1 (***B***) and ventral CA1 (***D***) recording session. Yellow bar denotes the median probability pattern depicted in ***C***, ***E***. ***C***, ***E***, Visualization of a single representative pattern from dorsal CA1 (***C***) and ventral CA1 (***E***). Points denote the approximate physical position of individual units. Closed circles denote active units (1), open circles denote inactive units (0), and gray circles denote units not included in the current 18-unit subsample. ***F***, Total entropy of dorsal CA1 populations is higher than that of ventral CA1 populations (*p* < 10^−6^, two-sided Wilcoxon rank-sum test, *n*_dCA1_ = 6000 subsamples from 6 recording sessions in 3 animals, *n*_vCA1_ = 4000 subsamples from 4 recording sessions in 2 animals). Subsample size of 18 units.

10.1523/ENEURO.0211-21.2021.f3-6 Extended Data Figure 3-6 Location of place fields along the virtual track. ***A***, Histogram of place field locations of all dorsal CA1 neurons. Yellow and grey dashed lines denote the 5th and 95th percentiles, respectively, of null distribution of place field centers obtained by temporally shuffling spike trains (*n* = 329 dorsal CA1 neurons). ***B***, Histogram of place field locations of all ventral CA1 neurons. Yellow and grey dashed lines denote the 5th and 95th percentiles, respectively, of null distribution of place field centers obtained by temporally shuffling spike trains (*n* = 196 ventral CA1 neurons). Download Figure 3-6, EPS file.

Taken together, these results suggest a coarsening of spatial representations from dorsal to ventral CA1 at the level of single neurons in virtual environments arose from differences in the activity of neurons when the animal was outside of their place fields. Consequently, the reduction of spatial information across the dorsal-ventral axis of CA1 appeared to be a universality principle of neuronal coding, the properties by which this coarsening of information occurred was different in real and virtual environments.

### Preserved pairwise correlations but weakened functional network structure in ventral CA1

The differences in single-neuron spatial encoding shown in Figure 3 are consistent with previous literature showing the heterogeneity of neuronal coding properties across the dorsal-ventral axis of CA1 (Jung et al., 1994; Ciocchi et al., 2015; Okuyama et al., 2016; Jimenez et al., 2018). However, these single-cell responses are embedded in a much larger population-level response, wherein functional interactions between individual neurons shape the collective activity of the CA1 population in ways that are critical for the encoding of position (Meshulam et al., 2017). We next wished to study how these functional interactions affected neuronal activity patterns in dorsal and ventral CA1 (Fig. 4*A*,*B*).

Although we found no significant differences in the mean pairwise correlations for dorsal and ventral CA1 (Fig. 4*C*,*D*), these averages masked the differences in the structure of correlations, as evidenced by the increased variance in dorsal CA1 correlations (Fig. 4*C*). To overcome this limitation, we represented the interactions between neurons not as mean values, but as network graphs, in which nodes denoted individual neurons and edges denoted a strong correlation between a pair of neurons (Fig. 4*E*,*F*). To understand how correlations were distributed across the population, we calculated the degree, or number of incident edges, for each node (Fig. 4*G*). Nodes in dorsal CA1 networks had significantly larger degrees than those in ventral CA1 networks, indicating that dorsal CA1 was more likely to contain highly connected “hub” neurons than ventral CA1. Importantly, regardless of the correlation threshold used to determine the presence of a network edge, the mean network degree of dorsal CA1 was larger than that of ventral CA1 (Fig. 4*H*). We next wished to examine whether this increased network connectedness led to an increased likelihood of coactive neurons in dorsal CA1, relative to ventral CA1. To do this, we took 18-unit random subsamples of each recorded population and plotted the number of coactive units in each 10-ms bin of the recording and found that dorsal CA1 populations were more likely to be highly coactive than those in ventral CA1 (Fig. 4*I*). Using this graph approach, we thus identified a novel difference in structure of correlations across neurons in the two regions, one that resulted in a more-connected network and more coactive neuronal population in dorsal CA1.

### Decreased dimensionality in ventral CA1 population activity

These data are consistent with previous studies that suggest that essential features of hippocampal representations of space are encoded in the joint activity of neurons and that these activity patterns cannot be easily summarized in single-neuron metrics such as place field size (Meshulam et al., 2017; Stefanini et al., 2020). To better understand how covariations between pairs of neurons shaped the dynamics of the broader populations, we used PCA (Luczak et al., 2009; Yu et al., 2009) to visualize low-dimensional representations of population activity in dorsal CA1 (53-neuron population) and ventral CA1 (48-neuron population; Fig. 5*A*). Each point in this space is the low-dimensional representation of the mean firing rate of every neuron in the population within a given time bin, which we defined as a state. This revealed complex trajectories corresponding to the evolution of population activity as the animal traversed the track. In both the dorsal and ventral CA1 examples, the population states occupied a different part of the space when the animal was running (indicated by the colored points) than when it was stationary (indicated by the gray points), consistent with the idea that behavior shapes neural activity (Vanderwolf, 1969; O’Keefe, 1976; O’Keefe and Recce, 1993; Skaggs et al., 1996; Foster and Wilson, 2006; Niell and Stryker, 2010; Dragoi and Tonegawa, 2011; Buzsáki, 2015; Villette et al., 2015; Vinck et al., 2015; Kay et al., 2016; Dadarlat and Stryker, 2017; Dipoppa et al., 2018; Kay and Frank, 2019; Chockanathan et al., 2020, 2021).

Although we observed complex trajectories of population activity in both dorsal and ventral CA1 in the same virtual environment (Fig. 5*B*), the fraction of principal components required to explain 80% of the variance in the data were larger for dorsal than ventral CA1 (Fig. 5*C*; Extended Data Fig. 5-1). Additionally, the fraction of the total variance explained by the top 25% of principal components, as well as the area under the variance explained curve, was larger for ventral CA1 (Fig. 5*D*,*E*). The population activity in ventral CA1 had fewer states, and therefore could be more easily summarized by a small number of variables than that of dorsal CA1. Taken together with previous results, this suggested that the ensemble activity of dorsal CA1 resided in a higher dimensional space than that of ventral CA1.

10.1523/ENEURO.0211-21.2021.f5-1 Extended Data Figure 5-1 Explained variance curves and corresponding nonlinear least squares fits. ***A***, Raw explained variance curves (black circles) and corresponding nonlinear least squares fits (blue lines; equations in inset) for each of the seven recording sessions in dorsal CA1. ***B***, Same as ***A*** for each of the six recording sessions in ventral CA1. Download Figure 5-1, EPS file.

### Decreased diversity and total entropy of population activity patterns in ventral CA1

The results of Figures 4, 5 indicate that the dimensionality of neural activity, and therefore the complexity of ensemble activity was larger in dorsal CA1 than ventral CA1. To understand what features of this activity conferred this increased complexity, we dissected the statistical properties of the population firing patterns in each region. To do this, we first defined a pattern of ensemble activity as a vector of neurons where an active cell was denoted with a 1 and an inactive cell denoted with a 0. In a given 10-ms window, then a pattern or state of activity was defined as a vectors of 1 and 0 s (Fig. 6*A*; Luczak et al., 2009; Miller et al., 2014; de Ruyter van Steveninck et al., 1997; Schneidman et al., 2006). We then compared the probability distributions of patterns from 18-neuron populations (made by subsampling from all recorded cells in a given recording session) in dorsal and ventral CA1 (Fig. 6*B*,*D*). As illustrated by these representative examples, the pattern probability distribution was broader in dorsal CA1 than in ventral CA1, indicating a more diverse set of patterns. This was consistent with our previous finding that the patterns that occurred in dorsal CA1 had more coactive neurons than those in ventral CA1 (Figs. 4*I*, 6*C*,*E*). We quantified the diversity of patterns by subsampling the population in each region using the entropy framework from statistical mechanics (see Materials and Methods). The higher diversity of patterns in dorsal CA1 was reflected in its increased total entropy, relative to ventral CA1 (Fig. 6*F*).

### Population activity in ventral CA1 is better explained by pairwise interactions than in dorsal CA1

The number of patterns explored by populations in either dorsal or ventral CA1 and the dimensionality of the neural code each reflect an underlying feature of CA1. A property such as functional connectivity, which reflects how neurons interact with one another, should constrain the states that are occupied by the populations as well as the dimensionality of the neural code. We wished to know whether we could predict the frequency of activity patterns observed in groups of neurons by simply looking at the functional interactions between pairs of neurons. Another way of studying this is to ask to what extent the global structure of the network can be completely defined by these pairwise interactions.

**Figure 7.**
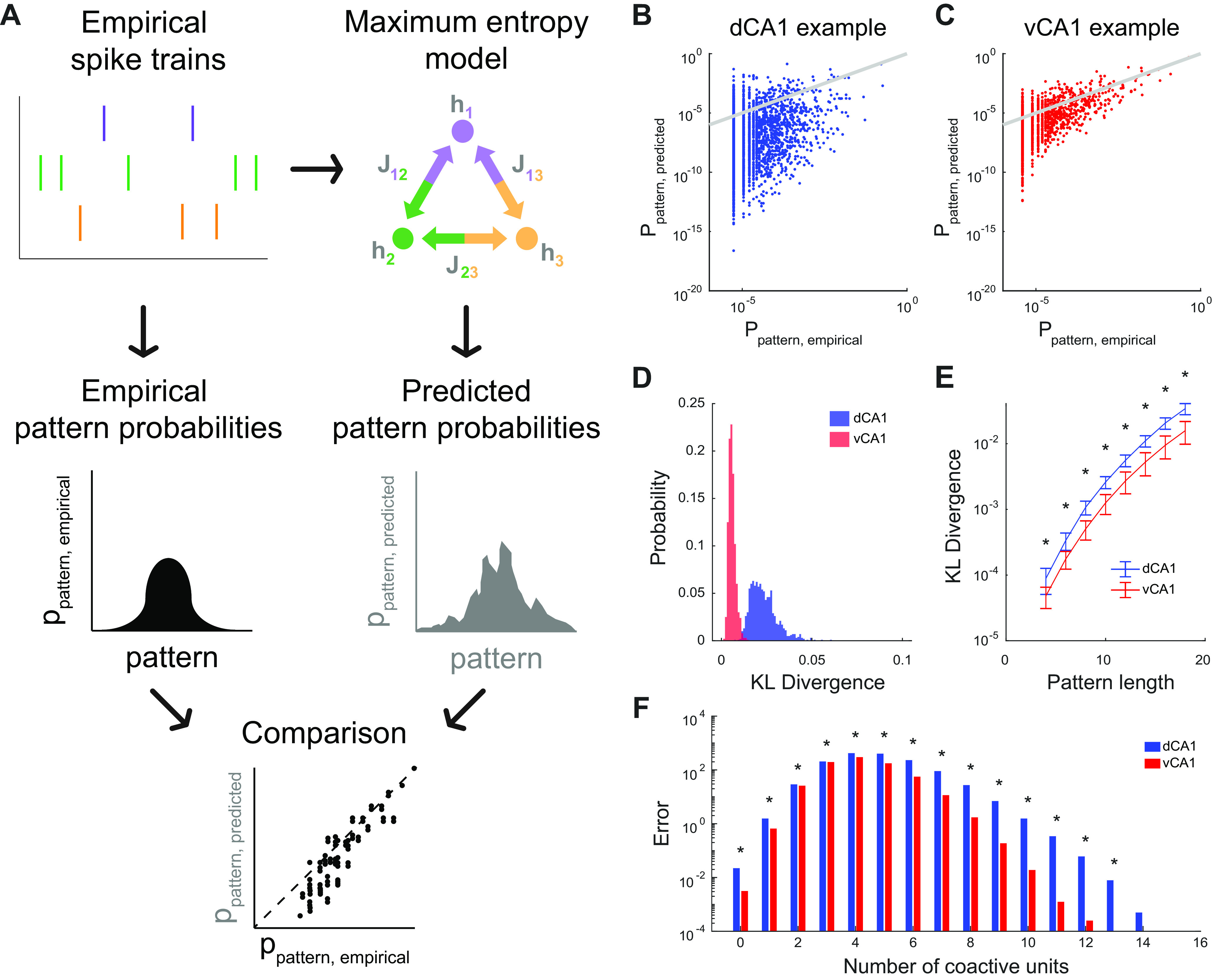
Pairwise maximum entropy models predict population patterns better in ventral CA1 than in dorsal CA1. ***A***, Schematic of maximum entropy models. Every neuron is assigned an activity term *h_i_* and every pair of neurons was assigned an interaction term *J_ij_*. Both terms were fit from the empirical spiking data. The models were then used to estimate the probability of every population pattern solely on the basis of these terms. The predicted pattern probabilities were then compared with the respective empirical probabilities. ***B***, ***C***, Empirical pattern probabilities and maximum entropy model predicted pattern probabilities for a representative 18-unit subsample of a dorsal CA1 (***B***) and ventral CA1 (***C***) recording session. Each point denotes a single pattern and gray line denotes unity. ***D***, Distributions of KLD generated from 1000 random samples of 18-unit subpopulations in a representative dorsal and ventral CA1 recording session. ***E***, KLD was larger for dorsal CA1 populations than ventral CA1 populations across a range of subpopulation sizes from 4 to 24 units. Asterisks denote significant difference between dorsal and ventral CA1 populations (for each pattern length: *p *<* *10^−6^, two-sided Wilcoxon rank-sum test, *n*_dCA1_ = 600 samples from 6 recording sessions in 3 animals, *n*_vCA1_ = 400 samples from 4 recording sessions in 2 animals). Error bars denote SEM. ***F***, Pattern probability prediction error of maximum entropy model, grouped by number of coactive units, generated using 1000 18-unit subsamples in each recording session. Asterisks denote significant differences between dorsal and ventral CA1 prediction errors (for each pattern type: *p *<* *10^−6^, two-sided Wilcoxon rank-sum test, *n*_dCA1_ = 6000 samples from 6 recording sessions in 3 animals, *n*_vCA1_ = 4000 samples from 4 recording sessions in 2 animals).

We approached this using the maximum entropy model, which explains the probability of all observed firing patterns of large populations of neurons with as little structure as possible (Fig. 7*A*). If, for example, pairwise interactions were sufficient to explain the global structure of population activity, then a second order maximum entropy model would be able to predict population activity patterns based only on the firing rates of single neurons, reflected in the *h_i_* terms, and on the functional interactions between pairs of neurons, reflected in the *J_ij_* terms (Schneidman et al., 2006; Shlens et al., 2006; Meshulam et al., 2017; Chockanathan et al., 2020). When we compared the predicted and empirical distributions as a scatter plot for a representative 18-neuron subsample (Fig. 7*B*,*C*), the maximum entropy model for the dorsal CA1 population showed many more points further away from the unity line than ventral CA1, indicating a higher error in prediction. Using the KLD a measure of the goodness of fit, we found that, across all pattern lengths we considered, the KLD was significantly larger for dorsal CA1 than ventral CA1 (Fig. 7*D*,*E*). Pairwise interactions were better able to predict the state of the overall population in ventral CA1 than dorsal CA1, suggesting that pairwise correlations had significantly less explanatory power in dorsal CA1 as compared with ventral CA1. This was despite the fact that the values of the *h_i_* and *J_ij_* parameters were not significantly different for the two regions (Extended Data Fig. 7-1), consistent with our previous observation that neither the mean firing rates nor the mean pairwise correlations were significantly different for dorsal and ventral CA1 (Figs. 3*J*, 4*D*). Thus, while Figures 5, 6 show how the population activity in dorsal CA1 is more complex and diverse than that of ventral CA1, the results of our maximum entropy models demonstrate that this increased dimensionality cannot be explained simply by changes the structure of firing rates and pairwise interactions.

10.1523/ENEURO.0211-21.2021.f7-1 Extended Data Figure 7-1 Maximum entropy parameters were similar for dorsal and ventral CA1. ***A***, Mean maximum entropy activity terms *h_i_* from 1000 samples of 18-unit subpopulations. There was not a significance in *h_i_* between dorsal and ventral CA1 populations (mean ± std: dCA1 = –3.322 ± 0.203, vCA1 = –3.737 ± 0.549, *p *=* *0.48, two-sided Wilcoxon rank-sum test, *n*_dCA1_ = 6 recording sessions, *n*_vCA1_ = 4 recording sessions). Each point denotes a recording session. ***B***, Mean maximum entropy activity terms *h_i_* across a range of subpopulation sizes from 4 to 18 units. For each pattern length, 1000 subsamples were performed per recording session. Error bars denote SEM. ***C***, Mean maximum entropy pairwise interaction terms *J_ij_* from 1000 samples of 18-unit subpopulations. There was not a significance in *J_ij_* between dorsal and ventral CA1 populations (mean ± std: dCA1 = 0.075 ± 0.136, vCA1 = 0.044 ± 0.036, *p *=* *0.48, two-sided Wilcoxon rank-sum test, *n*_dCA1_ = 6 recording sessions, *n*_vCA1_ = 4 recording sessions). Each point denotes a recording session. ***D***, Mean maximum entropy pairwise interaction terms *J_ij_* across a range of subpopulation sizes from 4 to 18 units. For each pattern length, 1000 subsamples were performed per recording session. Error bars denote SEM. Download Figure 7-1, EPS file.

Instead, these data suggested that, in dorsal CA1, groups of neurons were co-active more often than would be predicted by their pairwise interactions. Indeed, when we then separated the patterns by the number of coactive units and analyzed the prediction error in each pattern class (Fig. 7*F*), we found that the prediction errors in dorsal CA1 were larger than those in ventral CA1. Importantly, this gap was especially pronounced for patterns with high numbers of coactive units, suggesting that such highly synchronous patterns occurred far more frequently than would be predicted from pairwise functional coupling. Consistent with this idea, other groups have shown that the coactivation of these groups of neurons arise from higher order interactions (Ohiorhenuan et al., 2010). Our data suggest that these higher order interactions contribute significantly to shape ensemble activity in dorsal CA1 but are relatively weak or absent in ventral CA1. Such high order interactions may arise from the increased number of hubs we observed in the functional network structure, thereby driving the increased dimensionality of dorsal CA1 population codes of space.

### Decreased population-level spatial information in ventral CA1

Our data thus far reveal an underlying link between the functional organization of activity and the statistical implications for that activity in terms of the dimensionality of neuronal codes along the dorsal-ventral axis of CA1. We finally wished to determine what these differences in the statistics of population codes might mean for the representation of space throughout the hippocampus. Previous work has demonstrated that the activity of dorsal CA1 populations can be used to encode and decode the position of an animal in an environment (Wilson and McNaughton, 1993; Ziv et al., 2013). While individual ventral CA1 neurons have lower spatial information, comparatively little is known about how this affects the activity of populations (Keinath et al., 2014), the efficiency of this population activity on encoding information about an animals position, and what, if anything, these different strategies reveal about the functional heterogeneity of the hippocampus. To address this gap, we used an information theoretic approach (de Ruyter van Steveninck et al., 1997) to calculate the spatial information of population patterns and compare these quantities between dorsal and ventral CA1.

For a neural population to represent multiple spatial positions in a cognitive map, the activity patterns must be diverse as the animal navigates its environment. For the map to be reliable, patterns must correspond, reproducibly, to the position of the animal. Two components shape the spatial information content of populations: (1) the total entropy, which reflects the total number of population patterns observed over all regions of space; and (2) the noise entropy, which reflects the population patterns observed in a specific region of space (for details, see Materials and Methods). The difference between these two quantities is the information of the population, which reflects the degree to which the occurrence of a particular pattern can be used to infer the animal’s spatial position.

**Figure 8.**
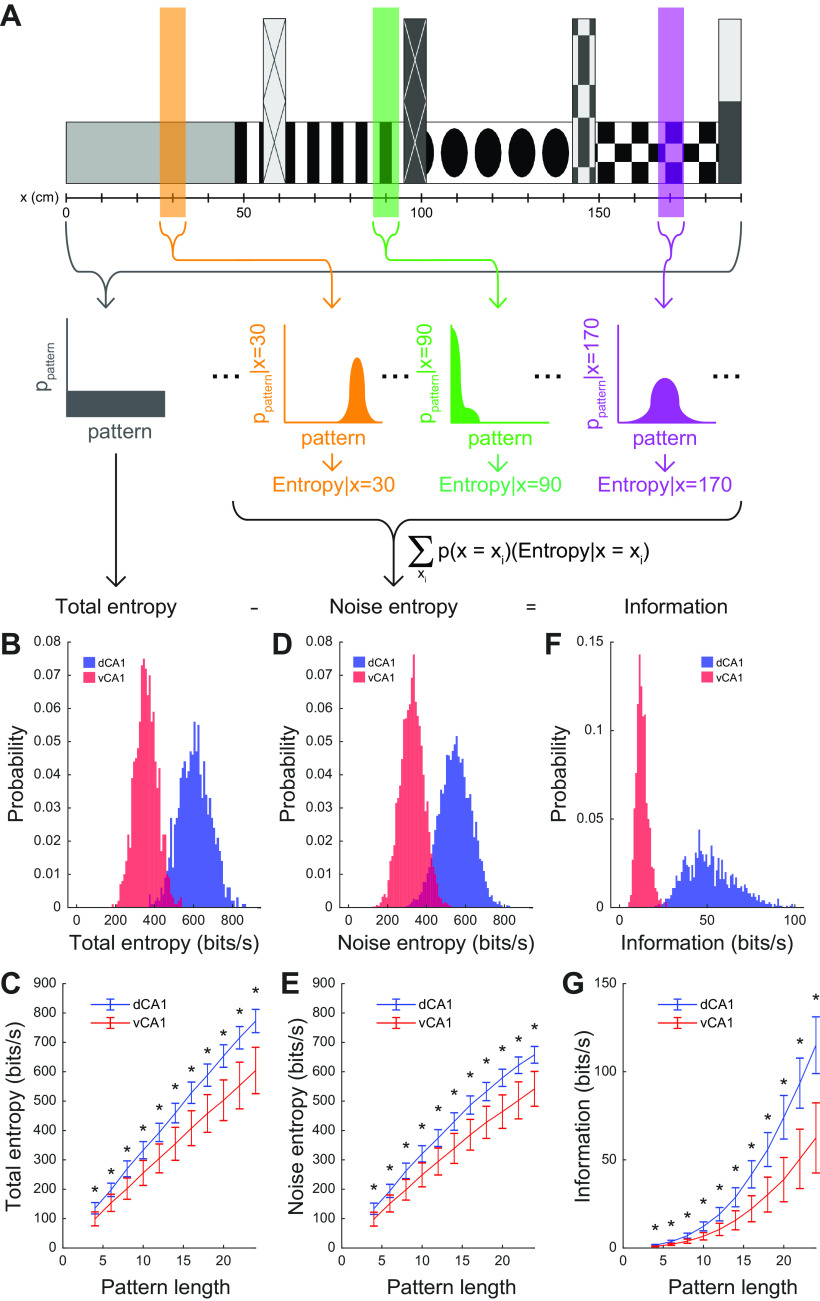
Dorsal CA1 populations collectively encode more spatial information than ventral CA1 populations. ***A***, Schematic for calculating spatial information of CA1 populations. The conditional pattern probability distributions (yellow, green, and purple) were generated from the population patterns that were observed when the animal was at a particular position on the virtual track. The overall pattern probability distribution (gray) was generated by pooling the probabilities of all observed patterns, regardless of the animal’s position. The entropy formula, $\sum^{}-p(log_{2}p)$ was then applied to each of the distributions. The total entropy was calculated from the overall distribution, while the noise entropy was calculated from conditional distributions, weighted by the amount of time the mouse spent at each position. The information was the difference between the total entropy and the information entropy. ***B***, Distributions of total entropy generated from 1000 random samples of 18-unit subpopulations in a representative dorsal and ventral CA1 recording session. ***C***, Total entropy was significantly larger for dorsal CA1 populations than ventral CA1 populations across a range of subpopulation sizes from 4 to 24 units. Asterisks denote significant difference between dorsal and ventral CA1 populations (for each pattern length: *p* <10^−6^, two-sided Wilcoxon rank-sum test, *n*_dCA1_ = 600 samples from 6 recording sessions in 3 animals, *n*_vCA1_ = 400 samples from 4 recording sessions in 2 animals). Error bars denote SEM. ***D***, Distributions of noise entropy generated from 1000 random samples of 18-unit subpopulations in a representative dorsal and ventral CA1 recording session. ***E***, Noise entropy was significantly larger for dorsal CA1 populations than ventral CA1 populations across a range of population sizes from 4 to 24 units. Asterisks denote significant difference between dorsal and ventral CA1 populations (for each pattern length: *p *<* *10^−6^, two-sided Wilcoxon rank-sum test, *n*_dCA1_ = 600 samples from 6 recording sessions in 3 animals, *n*_vCA1_ = 400 samples from 4 recording sessions in 2 animals). Error bars denote SEM. ***F***, Distributions of spatial information generated from 1000 random samples of 18-unit subpopulations in a representative dorsal and ventral CA1 recording session. ***G***, Spatial information was significantly larger for dorsal CA1 populations than ventral CA1 populations across a range of population sizes from 4 to 24 units. Asterisks denote significant difference between dorsal and ventral CA1 populations (for each pattern length: *p *<* *10^−6^, two-sided Wilcoxon rank-sum test, *n*_dCA1_ = 600 samples from 6 recording sessions in 3 animals, *n*_vCA1_ = 400 samples from 4 recording sessions in 2 animals). Error bars denote SEM.

We generated pattern probability distributions conditioned on the animal’s position (Fig. 8*A*) and quantified their diversity using entropy. A high entropy denoted a broad, flat probability distribution in which many patterns occurred with roughly equal probabilities while low entropy denoted a narrow distribution dominated by a small number of patterns.

We found that population spatial information was higher in dorsal CA1 than in ventral CA1 (Fig. 8*F*,*G*), suggesting that the asymmetric representation of space found at the single unit level (Fig. 3*H*) is preserved in populations. This increased information in dorsal CA1 was underpinned by a larger total entropy, suggesting that the overall number and diversity of observed patterns was larger in dorsal CA1 (Fig. 8*B*,*C*), consistent with our previous results (Figs. 4-6). Interestingly, however, we found that the noise entropy was also larger in dorsal than ventral CA1, suggesting that the diversity of patterns was larger in dorsal CA1 even within a single region of space (Fig. 8*D*,*E*). Additionally, we calculated the entropy conditioned on the animal’s behavioral state and found that while entropy increased with running in dorsal CA1, it decreased slightly with running in ventral CA1 (Extended Data Fig. 8-1).

10.1523/ENEURO.0211-21.2021.f8-1 Extended Data Figure 8-1 Changes in entropy with running. ***A***, ***B***, Scatter plot of entropy conditioned on stationary periods versus entropy conditioned on running periods in dorsal CA1 (***A***) and ventral CA1 (***B***; *n*_dCA1_ = 600 subsamples from 6 recording sessions, *n*_vCA1_ = 400 subsamples from 4 recording sessions). Grey line denotes unity. Each point denotes a subsample. ***C***, ***D***, Distribution of entropy conditioned on run velocity in dorsal CA1 (***C***) and ventral CA1 (***D***). Each color denotes the distribution of entropy values from a given 5 cm/s-width bin (*n*_dCA1_ = 600 subsamples from 6 recording sessions for each bin, *n*_vCA1_ = 400 subsamples from 4 recording sessions for each bin). Black squares denote the average entropy value for each bin. Each point denotes a subsample. Download Figure 8-1, EPS file.

The findings of increased dimensionality shown in Figures 5, 6 indicated that dorsal CA1 has a larger number of population patterns. The results in Figure 8 show that dorsal CA1 uses this increased neural “vocabulary” to represent space more effectively than ventral CA1. Taken together, our results provide additional evidence for the model of a functionally heterogeneous hippocampus, with dorsal CA1 specializing in spatial cognition as compared with ventral CA1.

## Discussion

We recorded the activity of neuronal populations in dorsal and ventral CA1 and examined the manner in which they collectively represented an animal’s position on a virtual track. Not only did we recapitulate earlier findings that individual neurons in ventral CA1 convey less information about position than those in dorsal CA1 (Jung et al., 1994), we also demonstrated that the collective activity of the population was differentially organized in the two regions. Dorsal CA1 populations formed more connected networks and showed more complex patterns of activity, which manifested as a larger total entropy, relative to ventral CA1 populations. This indication of increased dimensionality in dorsal CA1 activity was further supported by our finding that pairwise interactions were better able to predict population-level firing patterns in ventral CA1 than in dorsal CA1. Finally, by examining how the activity patterns in each region were organized by the position of the animal, we found that the population-level spatial information was also higher in dorsal CA1 than ventral CA1.

It should be noted that all the mice included in this study were males. Sex is a critical biological variable and should be considered in study design (Shansky, 2019). In a previous study of head-fixed wheel running similar to the task used in this experiment, it was found that female animals behaved differently on a run wheel, including how they adapted and habituated to the wheel while being head fixed (Warner and Padmanabhan, 2020). These sex differences may be related to anxiogenic behaviors, and could influence activity patterns in ventral CA1, as has been previously shown (Ciocchi et al., 2015; Jimenez et al., 2018). As such, this critical question goes beyond the scope of this study but is the focus of future work.

Our results should be placed in the context of a key prior study, which found that spatial coding across dorsal and ventral CA1 was largely conserved at the population level, although place fields of individual neurons were larger and less informative in ventral than dorsal CA1 (Keinath et al., 2014). While that study performed recordings in real-world 2D open-field environments using chronically implanted electrodes, in our study, we employed acute head-fixed recordings in a virtual 1D track. Virtual environments approximate several features of real-world navigation, including distal visual cues, optic flow, and proprioceptive cues. However, the head-fixed virtual reality setup in our study did not allow for motion cues from vestibular inputs or air flow. Additionally, real world environments have spatial variations in olfactory, tactile, and auditory properties that are largely absent in virtual environments (Minderer et al., 2016). Although the degree to which these different sensory inputs contribute to spatial coding is still an area of active investigation, it is reasonable to expect that virtual reality environments create different demands on the spatial coding system than real-world environments. Indeed, previous experiments of recordings in both virtual and real environments have found significant differences in place cell activity and theta frequency modulation (Ravassard et al., 2013; Aghajan et al., 2015). The methodological differences between our study and Keinath et al. (2014) may therefore explain some of the differences in our findings of place cell and population activity across the hippocampal axis. Despite this, other critical features of hippocampal function, such as the reductions in the spatial information in single neurons and the dimensionality differences in neuronal coding across the dorsal ventral axis of the hippocampus, point to principles of coding that are preserved in both real and virtual environments.

Along the dorsal-ventral axis of the hippocampus, there is a differentiation of functions that vary both in degree (for example, larger place fields in ventral CA1 than in dorsal CA1) as well as in kind (dorsal hippocampus is implicated in spatial memory, while ventral hippocampus is involved in social cognition and affect). These myriad cognitive and emotional roles are thought to arise from the variations in genetic (Thompson et al., 2008; Cembrowski et al., 2016), biophysical (Dougherty et al., 2012; Malik et al., 2016), circuit (Swanson and Cowan, 1977; Cenquizca and Swanson, 2007; Meira et al., 2018; Padmanabhan et al., 2019), and computational infrastructure (Jung et al., 1994; Kjelstrup et al., 2008; Ciocchi et al., 2015; Okuyama et al., 2016; Jimenez et al., 2018) across the dorsal-ventral axis of the hippocampus (Moser and Moser, 1998; Fanselow and Dong, 2010). Our observation of decreased collective encoding of space in ventral CA1 mirrors broader topographic variations in spatial coding throughout the hippocampal formation. For grid cells, which have periodic spatial firing maps, the spacing and size of the grid vertices increase from the dorsal to the ventral boundaries of the medial entorhinal cortex (MEC; Hafting et al., 2005; Stensola et al., 2012). A similar tuning gradient has been observed for head direction cells in Layer III of the MEC, with dorsal neurons exhibiting sharper directional firing fields than ventral neurons (Giocomo et al., 2014). As part of the canonical trisynaptic loop, CA1 receives indirect inputs from entorhinal cortex as well as direct afferents from CA3. This circuit is organized in a lamellar fashion, such that dorsal or ventral entorhinal cortex preferentially projects to dorsal or ventral CA3, respectively, which in turn preferentially projects to dorsal or ventral CA1 (Andersen et al., 1971, 2000; Sloviter and Lømo, 2012). Our data suggest that, in addition to receiving differential inputs, the differences in functional interactions between neurons within dorsal and ventral CA1 may evidence the diverse ways in which spatial representations are encoded in the hippocampus.

This diversity may confer various computational benefits that support flexible behavioral strategies across diverse ethological environments. For instance, if place fields were fixed in size, then, as the territory an animal explores increased, more neurons would be required to comprehensively represent this expanding space. Indeed, previous studies in dorsal CA1 have demonstrated that as environments are expanded, more place cells are recruited and individual place cells show more numerous and enlarged place fields (Fenton et al., 2008). An increase in the size of the environment may require additional features such as hedonic value be represented in the hippocampus, so as to organize and structure an increasingly large environment. The larger place fields and coarser representations of ventral CA1 may be better suited for these parallel tasks. Studies of rodents engaged in goal-directed spatial navigation tasks have found that place fields cover goal-related regions more densely than other regions (Hollup et al., 2001; Fyhn et al., 2002; Dupret et al., 2010; Xu et al., 2019; Sato et al., 2020), while another study identified a dedicated population of neurons that are exclusively active when the animal visits a rewarded region (Gauthier and Tank, 2018). The variation in spatial coding resolution between dorsal and ventral CA1 could provide the flexibility to broadly map a large environment and simultaneously represent salient regions with very high precision (Keinath et al., 2014). Future experiments that compare spatial representations in dorsal and ventral CA1 while animals explore multiple environments (real and virtual) that vary in size and saliency would allow these hypotheses to be explicitly tested.

While we have focused on the hippocampal representation of space as an animal navigates a virtual environment, neurons in CA1 encode an array of non-spatial variables. In dorsal CA1, individual neurons have been identified that respond to time (Manns et al., 2007; Pastalkova et al., 2008; MacDonald et al., 2011), velocity (Czurkó et al., 1999; Góis and Tort, 2018), head direction (Acharya et al., 2016; Leutgeb et al., 2000; Stefanini et al., 2020), and event sequences (Sun et al., 2020). Ventral CA1 neurons also exhibit diverse tuning properties for affective and social variables, including anxiety (Ciocchi et al., 2015; Jimenez et al., 2018) and conspecific identity (Okuyama et al., 2016; Deng et al., 2019; Rao et al., 2019). Often, neurons in both regions fire in response to combinations of multiple features (Ciocchi et al., 2015; Omer et al., 2018; Haimerl et al., 2019; Stefanini et al., 2020). Experimental and computational studies, both in hippocampus and primary sensory regions, such as the visual system and the main olfactory bulb, suggest that functional heterogeneity and mixed selectivity can increase the amount of information encoded by a population (Warland et al., 1997; Shamir and Sompolinsky, 2006; Padmanabhan and Urban, 2010; Tripathy et al., 2013; Fusi et al., 2016; Bernardi et al., 2020). Previous studies have demonstrated how this heterogeneity can improve the precision of spatial coding (Stefanini et al., 2020).

The diversity of population patterns we found in dorsal CA1 while the animal ran on the virtual track suggests that the activity of groups of neurons is marshalled in a way that is greater than the sum of their pairwise interacting parts to represent space. In parallel, in ventral CA1, the smaller number of patterns and lower spatial information may free up its coding “bandwidth” to represent other ethologically salient aspects of the world. We hypothesize that by differentially coordinating the activity of dorsal and ventral CA1 populations, the functional architecture of hippocampus supports distinct coding strategies, employing the coding space of dorsal CA1 to represent the animal’s position and leaving the capacity of ventral CA1 available for other emotive or affective behaviors.

## Synthesis

Reviewing Editor: Jonathan Lee, University of Birmingham Decisions are customarily a result of the Reviewing Editor and the peer reviewers coming together and discussing their recommendations until a consensus is reached. When revisions are invited, a fact-based synthesis statement explaining their decision and outlining what is needed to prepare a revision will be listed below. The following reviewer(s) agreed to reveal their identity: NONE.

We commend authors for their thorough and thoughtful consideration of the reviewer points. The 12-18 hour wait since anesthesia is sufficient, and as such we agree that no more data are required.

One remaining, very minor concern is that for the histology added to Figure 1 (thank you for adding this), the 3 probe locations shown in the small insets for ventral CA1 look like they may terminate in ventral stratum radiatum, rather than the ventral CA1 pyramidal layer (though it is difficult to tell). It is, therefore, somewhat unclear to the reviewers and future readers where single units were recorded. The authors may want to add another color asterisk to the estimated recording location where cells were identified on the probe, rather than just the probe tip. If this is not possible, please provide a brief explanation in your response.
